# Breastfeeding in Iran: prevalence, duration and current recommendations

**DOI:** 10.1186/1746-4358-4-8

**Published:** 2009-08-05

**Authors:** Beheshteh Olang, Khalil Farivar, Abtin Heidarzadeh, Birgitta Strandvik, Agneta Yngve

**Affiliations:** 1 Unit for Public Health Nutrition, Department of Biosciences and Nutrition, Karolinska Institute, Stockholm, Sweden; 2 Kermanshah University of Medical Sciences, Kermanshah, Iran; 3 Breastfeeding Office, the Ministry of Health IR of Iran, Tehran, Iran; 4 Guilan University of Medical Sciences, Rasht, Iran; 5 Department of Pediatrics, Institute of Clinical Sciences, Gutenberg University, Gutenberg, Sweden; 6 Akershus University College, Lillestrom, Norway

## Abstract

**Background:**

The need to promote breastfeeding is unquestionable for the health and development of infants. The aim of this study was to investigate prevalence, duration and promotion of breastfeeding status in Iran with respect to the Baby Friendly Hospital, government actions and activities by the Breastfeeding Promotion Society including comparison with European countries.

**Methods:**

This retrospective study is based on data from 63,071 infants less than 24 months of age in all the 30 urban and rural provinces of Iran. The data of breastfeeding rates were collected in 2005-2006 by trained health workers in the Integrated Monitoring Evaluation System in the Family Health Office of the Ministry of Health to evaluate its subordinate offices. A translated version of a questionnaire, used to assess the current breastfeeding situation in Europe, was used.

**Results:**

At a national level, 90% and 57% of infants were breastfed at one and two-years of age, respectively. Exclusive breastfeeding rates at 4 and 6 months of age at national level averaged 56.8% and 27.7%. Exclusive breastfeeding rates at 4 and 6 months of age in rural areas were 58% and 29%, and in urban areas 56% and 27%, respectively. The policy questionnaire showed that out of the 566 hospitals across the country 466 hospitals were accredited as Baby Friendly Hospitals, covering more than 80% of the births in 2006. A national board set standards and certified pre-service education at the Ministry of Health. Iran officially adopted the WHO International Code of Marketing of Breast Milk Substitutes in 1991. The legislation for working mothers met the International Labour Organization standards that cover women with formal employment. The Ministry of Health and Breastfeeding Promotion Society were responsible for producing booklets, pamphlets, breastfeeding journal, CD, workshops and websites. Monitoring of breastfeeding rates was performed every four years and funded by the Ministry of Health within the budgets assigned to the health care system.

**Conclusion:**

In comparison to many European Union countries, Iran showed a favorable situation in terms of breastfeeding rates and promotion of breastfeeding. Iran still needs to increase the rate of exclusive breastfeeding during the first six months.

## Background

The need to promote and support breastfeeding is unquestionable for the health and development of infants. It represents a public health priority everywhere, as confirmed by the Global Strategy on Infant Young Child Feeding, unanimously approved by the 55^th ^World Health Assembly (WHA) in 2003 [1].

Breastfeeding provides all essential nutrients for the first 6 months of life. Breastfeeding plays an important role in ensuring food security for a large proportion of babies in the world, where food security is defined as having enough food to maintain a healthy and productive life today and in the future [2]. Breast milk contains the long chain polyunsaturated fatty acids which are especially important for the development of the brain and the nervous system [3]. Breastfeeding is also associated with a decreased risk for many early-life diseases [4].

Low rates and early cessation of breastfeeding have important adverse effects on health, social and economic implications for women, children, the community and environment, and results in greater expenditure on national health care provision [5].

### Health in Iran

Iran is an Asian country with 30 provinces spread over an area of about 1,648,000 km^2 ^and has about 70 million inhabitants of whom 62% are living in urban and 38% in rural areas. According to UNICEF statistics in 2008 [6], the total fertility rate was 2 births per woman in 2006. The infant mortality rate was 30 per 1000 live births and the mortality rate in the population less than 5 years of age was 34 per 1000 in 2006 [6]. It seems that child mortality is going down over time and compared with other countries in the East Mediterranean region Iran had a good position [7].

The average annual growth rate of the urban population was 4.9% during 1970-1990 and 2.5% during 1990-2006. The gross national income per capita was US$3,000 in 2006. There were 850 active hospitals in Iran of which 566 had delivery and neonatal wards. In the urban areas 500 Health Centers provide health care and in the rural areas this is provided by 18,000 Health Houses. Iran has 41 Universities or Faculty of Medical Sciences (UFMS), all of them under jurisdiction of the Ministry of Health (MOH).

The capital province Tehran, has 12 million inhabitants and is divided into three geographic regions, covered by three different Universities of Medical Sciences (UMS) (Tehran 1 = Iran UMS, Tehran 2 = Tehran UMS and Tehran 3 = Shahid Beheshti UMS).

### The breastfeeding situation in Iran

The National Committee of Breastfeeding Promotion was established at the MOH in 1991 by the Minister of Health at that time, Professor Alireza Marandi [8]. The major strategy of the committee was to promote breastfeeding for preventing morbidity and mortality in infants and to progress their health.

The mission of the Children's Health Office in the MOH was reviewed in 2004 [9]. It included 10 objectives: to decrease growth abnormalities in children due to malnutrition, decrease mortality rates in both neonates and infants, decrease mortality rate in children under 5 years, decrease mental and physical growth abnormality, increase the rate and duration of breastfeeding within the first two years of life, increase the percentage of exclusive breastfeeding in infants up to 6 months, increase the percentage of complementary feeding in 6-9 months children, prescribe vitamin A and D and iron supplements and increase the number of Baby Friendly Hospitals (BFHs). Exclusive breastfeeding is officially recommended in Iran until six months of age [9], with the addition of vitamin A and D from two weeks after birth.

**Figure 1:**
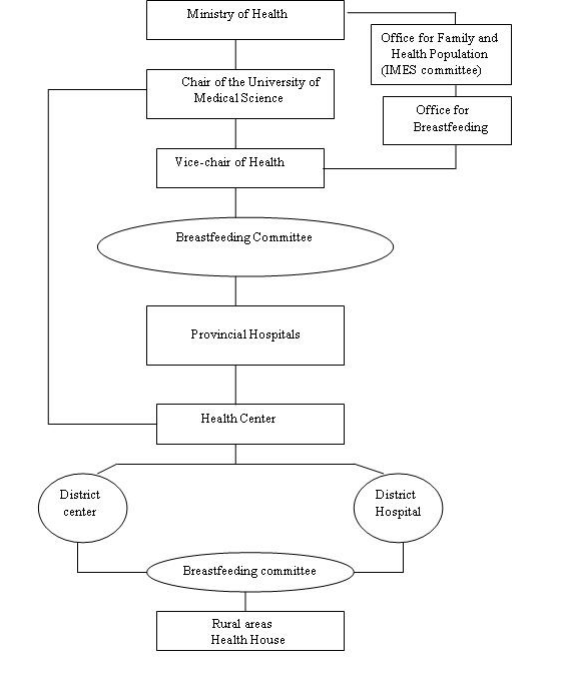
**Health System Network and breastfeeding promotion organizational structure in Iran**.

Each district in a province had a Health Network in which there was a breastfeeding committee. All committees at district level were supervised by the main breastfeeding committee at the UFMS, located in the capital city of the province (Figure 1). Child health was monitored through regular visits at Health Centers and Health House visits from the first week of life until 24th month of age. Child health monitoring was continued until 72 months after birth. At these visits the child was weighed, length and head circumference measured and the general health status assessed. The vaccination programme was followed. Iran also has a private health sector but vaccination is performed by the MOH in more than 95% of children.

The exclusive breastfeeding rates up to 6 months in Middle East-North African (MENA) region in the period of 2000 to 2006 were 28% [6]. In the MENA region, exclusive breastfeeding rates in Pakistan, Iraq, Saudi Arabia, Egypt and Iran were 16%, 25%, 31%, 38% and 44%, respectively [6].

Based on Demographic Health Survey (DHS) statistics in 2000 about 90% of the infants in Iran received any breastfeeding, while exclusive breastfeeding at 6 months was less than 45%. The exclusive breastfeeding rate at 6 months was about 44% in 2000 and decreased to 27% in 2004 [9].

This study intends to clarify the current situation (2006) regarding breastfeeding rates as well as breastfeeding promotion and support in Iran, as a first measure to design appropriate measures to stop the downwards trend in breastfeeding. This paper also reports on a survey of the implementation of breastfeeding policies in Iran.

## Methods

Exclusive breastfeeding is defined as providing only breast milk and no other liquids or solids except for those containing vitamins, minerals or medicines to the baby from birth [10,11]. Exclusive breastfeeding is considered superior at least until an infant is six months of age [10,12].

Breastfeeding rates were studied, using the Integrated Monitoring Evaluation System Survey (IMES) which collects data regarding the content and consequences of provision and delivery health services for promoting health in the country. The study population was obtained with regard to the general objective of IMES and on the basis of data required for Family and Population Health Office of the MOH in 2005-2006. The study population was based on data from IMES collected as follows: all mothers who had given birth at hospitals or other health facilities, all mothers who had taken their infants for the first vaccination at one of the rural or urban centers, all infants less than 2 years of age who lived in urban or rural areas and had been referred to Healthcare Center or Health House to receive healthcare, vaccination or treatment for any disease. It means that for exclusive breastfeeding rate data, only those infants were included who were between 1 day and 6 months of age. However, for general breastfeeding data, all infants were included (Table 1).

**Table 1:** Number and percentage distribution of the study population; data from Integrated Monitoring Evaluation System

**Age, months**	**Total number**	**Urban/rural** **%**	**Proportion** **distribution**	**Cumulative %**
0-4	2186	62/38	4.4	4.4
> 4-6	6248	61/39	10.8	15.2
> 6-9	15833	56/44	20.5	35.7
> 9-12	5290	54/46	9.3	45
> 12-18	17159	58/42	28	73
> 18-24	16355	56/44	27	100

Total	63 071	57/43	100	100

The data were gathered in urban and rural areas by health workers who were trained in the specifics of collecting health data including the definition of exclusive breastfeeding in the previous 24 hours. IMES data collection was performed in all rural areas through direct visits by trained interviewers who were employees of the MOH at UFMS and were sufficiently familiar with concepts and guidelines of provision of healthcare as well as asking the IMES questions through a questionnaire (see below). The questionnaire was validated in a pilot study in late 2004. Answers to questions about feeding patterns in infants at 4, 6 and less than 24 month of age were made by mothers and were not the reviewers' opinion. Questions were about the name of province, district and health center, number of household, mother's age and education, birth date, infant's age, weight, length and head circumference, breastfeeding exclusively and any breastfeeding. The data were collected from 15 September 2005 to 15 January 2006. In this paper only breastfeeding data are reported.

### Sampling method and sample size (IMES)

We used a multistage sampling method with three kinds of sampling:

1. Random cluster sampling with non-equal clusters. In each district we identified 20 clusters by random sampling from electrical power counters number list. Then two children were selected from each cluster to enter the study.

2. Sampling in the urban areas was from clients who came to Health Centers for vaccination (convenience sampling).

3. Random systematic sampling was used to sample infants in rural areas. The sampling frame was the list of childcare users which covered more than 99% of the infants in rural area.

### The policy questionnaire

In order to assess the current situation in regard to breastfeeding protection, promotion and support, a questionnaire (available from the first author upon request) was used during February to May 2007. This was a translation of one used in Europe [5,13]. The questionnaire was sent to the central committee of breastfeeding at the MOH, where it was completed based on national data in 2006. The questionnaire was used to investigate how the following topics were implemented in Iran as a whole and in its provinces:

1. Breastfeeding policy and/or action plans

2. Leadership for breastfeeding promotion

3. Training of health care staff

4. Baby Friendly Hospital Initiative (BFHI) [14]

5. Endorsement of the WHO International Code of Marketing for breast milk substitutes [15]

6. Legislation for working mothers

7. Community outreach, including mother support

8. Information, education, communication

9. Monitoring of breastfeeding duration and exclusiveness

10. Activities directed towards disadvantaged groups

### Statistical analysis

IMES data were analyzed using STATA version 8.0 and Survey Analysis commands by using each medical university as a separate strata and each data collection area as primary sampling unit (PSU) and proportion of sampled person to population of under 2 years of age infants as weight. Breastfeeding rates were calculated with 95% confidence interval (CI).

### Ethical approval

Approval to conduct this analysis was obtained from the ethics committee at the MOH in Iran in February 2007.

## Results

The number, age and the distribution of participants in the IMES are shown in Table 1. The proportions between urban and rural provinces are shown, indicating that the study population was representative for Iran.

### Breastfeeding rates (IMES)

At a national level, a mean of 90% (95%CI: 79.3, 96.1) and 57% (95%CI: 33.1, 91.8) of infants had been breastfed between 12 to 15 months and 20 to 23 months, respectively (Additional file 1; Table S1). The exclusive breastfeeding rates were 57% (95%CI: 38.8, 79.6) at 4 months and 28% (95%CI: 15.4, 52.7) at 6 months. The differences in exclusive breastfeeding rates at 4 and 6 months between regions are shown in Additional file 1; Table S1.

**Figure 2:**
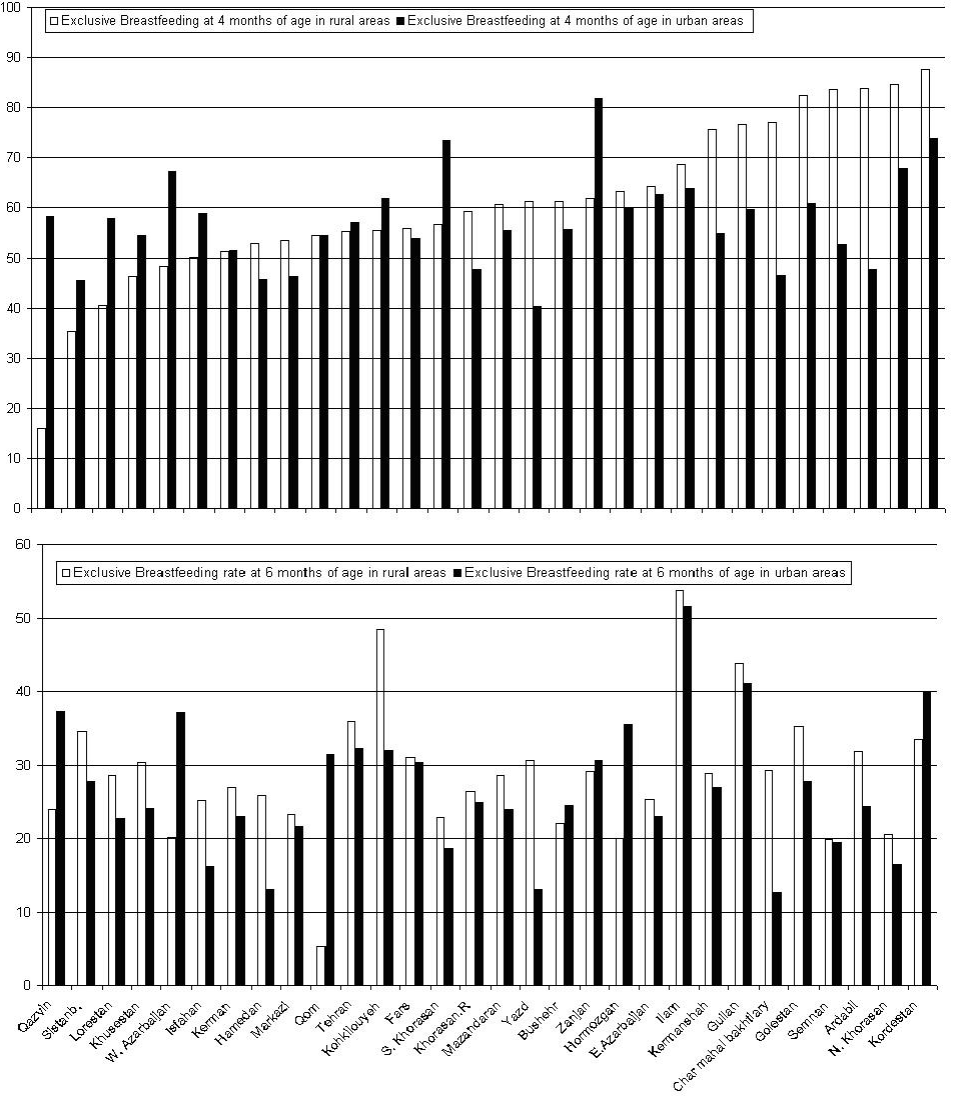
**Exclusive breastfeeding rates at 4 and 6 months of age in rural and urban areas, Data from Integrated Monitoring Evaluation System**.

The exclusive breastfeeding rates in rural areas were 58% (95%CI: 15.9, 87.6) and 29% (95%CI: 5.3, 53.8) at 4 and 6 months of age respectively, and corresponding figures in urban areas were 56% (95%CI: 40.3, 81) and 27% (95%CI: 12.7, 41.1) (Additional file 1; Table S1 and Figure 2).

The exclusive breastfeeding rates at 4 and 6 months of age and their confidence intervals are presented in Additional file 1; Table S1. The results vary from 30% to 94% at 4 months of age and 13% to 55% at 6 months of age. The regions with the lowest exclusive breastfeeding rate at 4 month of age were Sistanblochestan, Qazvin and Yazd with low, middle and high socioeconomic status (SES), respectively. The lowest exclusive breastfeeding rate at 6 month of age was seen in Yazd, Isfahan and North Khorasan with high SESs. The highest rates could be seen in North Khorasan (high SES), Kordestan (low SES) and Ardebil (low SES) at 4 month of age. The highest rate of exclusive breastfeeding at 6 month of age was in Ilam (low SES), Guilan (middle) and Kohkilouyeh (low SES) (additional file 1; Table S1). These results indicate that socioeconomic status is not the most important factor for mothers to breastfeed.

### Policy, planning and management

All provinces and UFMS are supposed to follow the national policy which is regulated by the MOH (Figure 1). Four objectives have been adopted by universities, including implementing the Ten Steps for Successful Breastfeeding and helping mothers to initiate breastfeeding soon after birth [16], to breastfeed exclusively for 6 months and to continue breastfeeding up to 2 years of age.

A national plan including general objectives and recommended strategies has been developed by the MOH [17]. Action plans including specific objectives, targets and activities have been developed by health workers in all provinces. All UFMS have a coordinator who should report these activities to the national coordinator at the MOH on a regular basis. Members of the provincial committee are all chairs and vice chairs of UFMS, experts from medicine, nursing and midwifery schools, pediatric nutritionists and gynecologists/obstetricians as well as nutrition departments in each province. The deputy of health at each UFMS coordinates these committees.

### Training

There is a national board that sets standards and certifies pre-service education at the MOH. The 18-hour UNICEF/WHO course on promotion of breastfeeding and BFH has been introduced by the MOH [18]. The 40-hour WHO/UNICEF course on breastfeeding counseling has been introduced by the MOH in all UFMS [19]. Most of these courses are endorsed and lead to credits. The training covers nurses, midwives, general physicians, pediatricians and obstetricians. Health workers have, in the majority of cases, completed the 18-hour course and are in the process to completing the longer course, since they need it to keep their positions. The course requirement in time is 36 hours for physicians and specialists.

### Baby Friendly Hospital Initiative

Each province has a BFHI coordinator in the provincial health center who reports to the Deputy of Treatment Affairs in each UFMS. The proportion of births covered by BFHs is more than 80 percent. All BFHs are evaluated annually by the Breastfeeding Office at the MOH. Eighty two percent (466/566) of hospitals across the country were accredited as BFHs in 2006. Additional file 1; Table S1 shows the number of BFHs by province and UFMS. The number of BFHs in each province varies from 3 to 72. The number of hospitals is related to the population in each district or province. The number of BFHs in relation to the population varied from 0.28 to 1.31 per 100,000 inhabitants (Additional file 1; Table S1). The two provinces with lowest numbers of BFHs per 100,000 population were Qom and Sistanbalochestan, with 0.28 and 0.33, respectively. The two highest were in Yazd and Golestan with 1.31 and 1.05, respectively (Additional file 1; Table S1). The proportion of BFHs in relation to total number of hospitals in the different provinces varied between 0.5 and 1. The two lowest proportions of BFHs in relation to total number of hospitals were seen in Qom and Kermanshah with 0.5 and 0.56, respectively (Additional file 1; Table S1).

### WHO International Code and subsequent relevant WHA resolutions

Iran officially ratified the WHO International Code of Marketing of Breast Milk Substitutes in 1991 [20] and also favored of the International Code of Marketing of Breast milk Substitutes and of subsequent relevant WHA resolutions. Iran adopted many provisions (e.g., no advertising of breast milk substitute to the public, no direct and indirect free samples or gifts to mothers or their relatives, no gifts or personal samples to health workers and etc) of the Code also in its directive for the internal market of infants and follow on formulas.

### Legislation for working mothers

The Maternity Protection act of the International Labor Organization (ILO) [21] sets standards for protecting and supporting breastfeeding in Iran only among governmental working mothers and working mothers who have insurance that is paid for by themselves or their employers. These standards include the provision of a minimum 24 weeks of paid maternity leave as well as the right to one paid breastfeeding break daily or reduction of work by one hour per day to promote longer duration of breastfeeding without loss of payment up to 24 months of age [22].

The ILO recommendations also state that maternity leave payments should be at least two-thirds of previous earnings. This is adhered to completely by government institutions in all provinces of Iran. There are day care centers and kindergartens next to most of the governmental offices. Maternity leave from work was extended from 16 to 24 weeks at the end of 2006. After six months, reduction of work by one hour per day is optional, in order to promote breastfeeding up to 24 months of age. It is important to note that for mothers without insurance, working outside governmental institutions, there is no paid maternity leave or right to breastfeeding breaks.

### Community outreach, including voluntary mother to mother support

The Iranian Breastfeeding Promotion Society (BFPS) [23] is a non-governmental, non-political and non-profit organization, which was established in 1991. This society supports mothers through a web site and telephone support service. There is no special organization for mother to mother support [23].

### Information, education, communication

The Breastfeeding Office at the MOH and BFPS (non-governmental) are responsible for producing pamphlets, booklets, leaflets, a breastfeeding journal, videos, CD, TV spots and web based information as well as workshops for staff. CD and videos are used for training mothers and physicians in BFHs. These materials are revised regularly by the National Committee of Breastfeeding. The World Breastfeeding Week activities are implemented in all provinces by the MOH and in Tehran is partially by UNICEF. There is also a governmental breastfeeding website [23].

### Monitoring

Monitoring of breastfeeding rates is performed every four years by the Breastfeeding Office at the MOH within the budgets assigned to the health care system.

### Disadvantaged groups

All of the women in Iran have the opportunity to use material for breastfeeding promotion from the MOH. There is a possibility for mothers without insurance to receive monetary support from Emdad Relief Committee, which is funded by public resources.

## Discussion

Three important findings in our study were the identification of the rates of exclusive breastfeeding at 4 and 6 months of age, and the high proportion of accredited BFHs. The policy questionnaire showed that the Iranian authorities have made many measures to improve mother and infant care. Despite those efforts there were large variations in breastfeeding rates, which were not easily explained.

Large provinces have UMS and small provinces have Medical Faculty. The differences in number of BFHs were due to difference in the number of hospitals in each Medical University or Medical Faculty (e.g. Jahrom district has only one Medical Faculty and one hospital which is a BFH). BFHs covered more than 80% of deliveries across the country in 2006. BFHs had applied the WHO breastfeeding definition.

The highest exclusive breastfeeding rate at 6 month of age was shown in Ilam, which is a small province with low SES and a more rural population. It could be explained by old traditions that support breastfeeding in that area. The proportions of BFHs in relation to total number of hospitals and BFH to 100,000 population in Ilam were 0.71 and 0.91, respectively. The highest exclusive breastfeeding rate at 4 month of age was in North Khorasan with a high SES and high education but the exclusive breastfeeding rate at 6 month of age had dropped off. It could be explained by the return to work by mothers. The proportions of BFHs in relation to total number of hospitals and BFH to 100,000 population in North Khorasn were 1.0 and 0.61, respectively. The lowest exclusive breastfeeding rate at 4 month of age was shown in Sistanbalochestan but it was not the lowest exclusive breastfeeding rate at 6 month of age. Sistanbalochestan has low SES and more rural and low educated population. There is seasonal movement in this area. The proportions of BFHs in relation to total number of hospitals and BFH to 100,000 population in Sistanbalochestan were 0.88 and 0.33, respectively. The lowest exclusive breastfeeding rate at 6 month of age was illustrated in Yazd with high SES, high education and early return to work. The proportions of BFHs and BFH in relation to total number of hospitals to 100,000 population in Yazd were 0.92 and 1.31, respectively. Qom had the lowest proportions of BFHs in relation to total number of hospitals and BFH to 100,000 population despite the exclusive breastfeeding rates at 4 and 6 months of age were 54.4% and 25.8%, respectively. Also the urban population was higher than the rural population in Qom province and the SES was high. The regions with low rates of exclusive breastfeeding rate at 4 and 6 months are characterized by low to middle SES and also by return to work, while the North Khorasan and Guilan with high rates can be seen as affluent parts of the country, Ilam and Kordestan are regarded as culturally more traditional and with a more rural population.

Higher rates of exclusive breastfeeding in rural areas compared with those in urban areas indicated that this situation could be due to tradition rather than socioeconomic status or high education. A cross-sectional study in the north district of Iran (Babol) in 1998 showed that the prevalence of breastfeeding was 87% and 89% at 12 months in urban and rural areas and 18% and 53% at 24 months, respectively [24]. In our results the prevalence of breastfeeding was 95% at 12 months in urban and rural areas and 66.7% and 33% at 24 months, respectively. On the other hand in this area in our study there is no clear relationship between high and low socioeconomic status, high and low proportion of BFHs and BFH to 100 000 population and the breastfeeding rates, suggesting that the breastfeeding rate is determined by many interactive factors.

Lower rate of exclusive breastfeeding in working mothers demonstrate the need to educate mothers, but the extension of paid maternity leave might be more important [25]. Other factors may be of importance and more research is required for identifying explanatory factors.

The exclusive breastfeeding rate at 6 month of age was about 44% in 2000 and decreased to 27% in 2004. Our findings support the decreasing trend since in this study nearly 28% breastfed at 6 month of age. The Breastfeeding Office became more active in late 2004, with support from the National Breastfeeding Committee. Iran had not had any International Board Certified Lactation Consultants (IBCLCs) until 2007 [26]. At the end of 2007, however, the first coordinator was appointed who was certified as an IBCLC. Furthermore paid maternity leave in Iran was increased from 16 to 24 weeks in 2007. Iran has a good family planning system; the growth rate of the population is under control and mothers are encouraged to work outside the home and have fewer children.

The status of BFH in March 2002 demonstrated that the number of Baby Friendly Hospitals in the world was 14,992, distributed over the eight regions [5]. The regions are West and Central Africa, Eastern and Southern Africa, Middle East and North Africa (MENA), East Asia and Pacific, South Asia, Americas and the Caribbean, Central and Eastern Europe and the Commonwealth of Independent States (CEE/CIS) and Industrialized Countries. Iran is located in the MENA region and had 376 BFHs in 2002. Thus Iran, as 1 out of 20 countries had 46% of the BFHs in this region. Iran was the first ranked country in terms of proportion of BFHs in the MENA region. Second and third ranked countries were Tunisia and Egypt, which had 17% and 15% of proportion of BFHs in MENA region, respectively. At the same time Saudi Arabia and United Arab Emirates had even fewer proportions of BFHs (0.25% and 0.5%, respectively). Despite the fact that Iran has a relatively good position from an international perspective; the MOH is planning to increase the number of BFHs. By comparison with some EU countries [5] Iran had an intermediate situation in regards to the number of BFHs (Table 2). Sweden had only a 34% exclusive breastfeeding rate at 6 months of age in 2000 despite 100% BFH indicating the complexity of the subject [5]. A recent review, regarding breastfeeding in Australia and Iran, showed that most Australian women discontinue breastfeeding before the recommended time [27]. The study attempted to identify variables influencing breastfeeding practices in Australia by comparing Australia with Iran, indicating comparatively high breastfeeding rate in Iran. Only approximately 4.5% of public and private hospitals in Australia [27] were BHF while the corresponding figure in Iran was 80%.

**Table 2:** BFH prevalence, percentage of births at BFH and exclusive breastfeeding (Excl. BF) prevalence in some EU countries [5] and Iran.

**Country**	**Year**	**BFHs/total hospitals**	**% of birth at BFHs**	**Excl. BF at 4 mo. (%)**	**Excl. BF at 6 mo. (%)**
Austria	1998	14/110	12	79	46

Germany	1998	18/1100	3	33	10

Italy	2000	7/700	1	47	13

Lithuania	2002	3/54	12	_	14

Portugal	1998	0/60	0	_	9

Sweden	2000	52/52	100	68	34

Iran	2006	466/566	≈80	57	28

The breastfeeding tradition is well established in Iran, and the history of breastfeeding can be traced back to the fourth century in the Canon Medicine Text Book which was written by Avicenna [28]. This important textbook was extensively used in European medical schools for centuries after Avicenna's death. In the Canon of Medicine, a chapter was allocated to the care of the newborn infant dealing with hygiene, breastfeeding and raising the child.

## Conclusion

The breastfeeding situation in Iran is good in comparison with several European countries and Australia. However, the exclusive breastfeeding prevalence had shown a downward trend at 4 and 6 months, which was confirmed in this new study. The results were far from meeting the WHO recommendation. The declining trend showed large big regional differences. Support for women needs considerable improvement in regards to the protection, promotion and support of breastfeeding, which would probably increase the national figures substantially.

## Competing interests

The authors declare that they have no competing interests.

## Authors' contributions

BO drafted the manuscript and coordinated the project between the MOH in Iran and Karolinska Institutet. KF was the head of Breastfeeding Office in the MOH and helped for preparing data collection in policy questionnaire. AB was responsible for IMES data and performed statistical analysis. BS helped to developing the study and writing the manuscript. AY participated in design of the study. All authors approved the final manuscript.

## Supplementary Material

Additional File 1 **Table S1**. Mean percentage of infants with exclusive breastfeeding (EXBF) and 95% confidence intervals (CI) at four and six months (m) of age and partial breastfeeding (BF) at one and two years of age in the different provinces in Iran. Click here for file
