# Supplementary material for: Breastfeeding in Iran: prevalence, duration and current recommendations
Source: Int Breastfeed J. 2009 Aug 5;4:8. doi: 10.1186/1746-4358-4-8 (PMC2734342; doi:10.1186/1746-4358-4-8)
Supplement: Additional File 1 — Table S1. Mean percentage of infants with exclusive breastfeeding (EXBF) and 95% confidence intervals (CI) at four and six months (m) of age and partial breastfeeding (BF) at one and two years of age in the different provinces in Iran. [file 1746-4358-4-8-S1.doc]

## Table S1. Mean percentage of infants with exclusive breastfeeding (EXBF) and 95 % confidence intervals (CI) at four and six months (m) of age and partial breastfeeding (BF) at one and two years of age in the different provinces in Iran.

| **Province** | **Population** | **UFMS**  **(n)** | **BFH/TotalH**  **n/n** | **BFH/**  **100000 population** | **EXBF at 4 m. Mean % (CI)** | **EXBF at 6 m.**  **Mean % (CI)** | **BF at 12-15 m. Mean %** | **BF at 20-23 mo. Mean %** |
| --- | --- | --- | --- | --- | --- | --- | --- | --- |
| Sistanblochestan | 2 405 742 | 2 | 8/9 | 0.33 | 36.8 (30-48) | 30.8 (27-35) | 86.7 | 45.3 |
| Qazvin | 1 143 200 | 1 | 7/7 | 0.61 | 43.2 (29-58) | 33.3 (31-36) | 91.4 | 52.8 |
| Yazd | 990 818 | 1 | 13/14 | 1.31 | 46.6 (39-54) | 15.4 (13-17) | 92.6 | 61.6 |
| Lorestan | 1 7165 27 | 1 | 9/9 | 0.52 | 47.6 (41-54) | 25.3 (23-27) | 95.1 | 64.6 |
| Hamedan | 1 703 267 | 1 | 14/14 | 0.82 | 49.1 (41-57) | 26.4 (24-29) | 94.5 | 91.8 |
| Markazi | 1 351 257 | 1 | 11/11 | 0.81 | 49.2 (41-58) | 22.2 (20-25) | 94.0 | 46.9 |
| Khorasan Razavi | 5 593 079 | 2 | 37/55 | 0.64 | 50.5 (45-56) | 25.4 (24-27) | 91.7 | 62.1 |
| Kerman | 2 652 413 | 2 | 18/21 | 0.67 | 51.4 (45-58) | 24.5 (23-26) | 82.4 | 54.9 |
| Khuzestan | 4 274 979 | 1 | 34/40 | 0.79 | 52.5 (46-59) | 25.4 (23-27) | 87.8 | 59.6 |
| Fars | 4 336 878 | 3 | 31/37 | 0.71 | 54.4 (50-58) | 30.6 (29-33) | 86.4 | 73.7 |
| Qom | 1 046 737 | 1 | 3/6 | 0.28 | 54.4 (40-70) | 25.8 (21-30) | 93.3 | 51.9 |
| Isfahan | 4 559 256 | 2 | 37/44 | 0.78 | 55.9 (51-61) | 17.9 (17-19) | 91.1 | 61.1 |
| Tehran | 13 000 000 | 3 | 72/97 | 0.55 | 56.7 (51-62) | 32.8 (31-35) | 88.8 | 51.0 |
| Mazandaran | 2 922 432 | 2 | 20/22 | 0.68 | 58.1 (50-66) | 25.8 (24-27) | 92.3 | 34.7 |
| Kohkiloyeboyrahmad | 634 299 | 1 | 4/6 | 0.63 | 58.2 (49-68) | 42.1 (39-45) | 91.2 | 64.5 |
| Kermanshah | 1 879 385 | 1 | 14/25 | 0.74 | 58.3 (51-66) | 27.4 (25-30) | 90.4 | 70.9 |
| Bushehr | 886 267 | 1 | 5/6 | 0.56 | 58.6 (51-66) | 23.5 (21-26) | 79.3 | 50.3 |
| Hormozgan | 1 403 674 | 1 | 9/9 | 0.64 | 60.6 (54-67) | 29.3 (27-32) | 88.6 | 33.1 |
| Semnan | 589 742 | 2 | 5/6 | 0.73 | 61.2 (51-71) | 19.5 (17-22) | 92.6 | 54.5 |
| East Azarbaijan | 3 603 456 | 1 | 21/25 | 0.58 | 63.3 (58-69) | 23.8 (22-26) | 86.0 | 53.4 |
| Charmahalbakhtiary | 857 910 | 1 | 5/5 | 0.58 | 63.5 (55-72) | 20.9 (19-23) | 92.4 | 42.3 |
| South Khorasan | 636 420 | 1 | 3/3 | 0.62 | 63.7 (55-73) | 21.3 (19-24) | 96.1 | 54.2 |
| Zanjan | 964 601 | 1 | 5/5 | 0.51 | 64.5 (57-62) | 30.0 (27-33) | 89.7 | 65.7 |
| Weast Azarbaijan | 2 873 459 | 1 | 18/19 | 0.62 | 64.9 (60-70) | 31.3 (29-33) | 86.0 | 54.4 |
| Ilam | 545 787 | 1 | 5/7 | 0.91 | 66.9 (59-75) | 52.7 (50-56) | 94.6 | 91.8 |
| Guilan | 2 404 861 | 1 | 18/22 | 0.74 | 68.3 (61-76) | 42.3 (40-44) | 84.1 | 66.6 |
| Golestan | 1 617 087 | 2 | 17/18 | 1.05 | 70.5 (66-75) | 31.7 (30-33) | 92.3 | 62.5 |
| Ardebil | 1 228 155 | 1 | 8/8 | 0.65 | 73.0 (65-81) | 27.6 (31-25) | 90.7 | 64.0 |
| Kordestan | 1 440 156 | 1 | 10/11 | 0.69 | 79.5 (75-84) | 37.3 (35-40) | 88.8 | 72.0 |
| North Khorasn | 811 572 | 1 | 5/5 | 0.61 | 76.6 (66-94) | 18.7 (16-22) | 93.4 | 40.5 |

Data from Integrated Monitoring Evaluation System and number of Baby Friendly Hospitals (BFHs) related to University or Faculty of Medical Sciences (UFMS) in Iran and data from Breastfeeding office in Ministry of Health (policy questionnaire)
